# “Psychiatry Perspectives in Neurocognitive Disorders” – Parallel Session from the 17^th^ International Summer School of Neurology

**DOI:** 10.25122/jml-2022-1028

**Published:** 2022-10

**Authors:** Stefana-Andrada Dobran, Alexandra Gherman, Dafin Fior Muresanu

**Affiliations:** 1 RoNeuro Institute for Neurological Research and Diagnostic, Cluj-Napoca, Romania; 2 Sociology Department, Babes-Bolyai University, Cluj-Napoca, Romania; 3 Department of Neuroscience, Iuliu Hatieganu University of Medicine and Pharmacy, Cluj-Napoca, Romania

Within the "17^th^ International Summer School of Neurology" event, on July 8^th^, the psychiatry session took place online, paralleling the neurology one, and being moderated by **Doina Cozman (Romania)**, Professor of Psychiatry and Clinical Psychology at the Department of Clinical Psychology from Iuliu Hatieganu University of Medicine and Pharmacy in Cluj-Napoca (Romania) and the President of the Romanian Association of Psychiatry and Psychotherapy. The proceeding focused on case studies of patients with diverse affections, mainly depression, cognitive impairment, and discussed the role of neurotrophic factors.

Prof. Dafin Muresanu, the EFNR President, delivered his thoughts on the 17^th^ year of intense collaboration, benefitting from a strong endorsement from the World Federation of Neurology (WFN), World Federation of Neurorehabilitation (WFNR), European Federation of NeuroRehabilitation Societies (EFNR), European Academy of Neurology (EAN), and many other organizations. Prof. Muresanu made a stance on working together on a significant topic at the European level, namely the concept of "brain health", which presumes a close collaboration between neurology and psychiatry, outlining his belief that the future would bring together the two disciplines due to the fantastic advances, with a strong accent being placed on the interdisciplinary approach.

The first session of the day introduced **Adela Ciobanu (Romania)**, Habilitated Professor of Psychiatry at Carol Davila University of Medicine and Pharmacy in Bucharest (Romania), and Head of 1^st^ Clinical Department at Professor Dr. Alexandru Obregia Hospital (Romania), presenting the management of treatment-resistant depression in the elderly. Prof. Ciobanu followed the case of a 75-year-old woman with a depressed mood, anxiety, and insomnia, describing behavioral traits, treatment history, and symptoms. A very in-depth presentation of the mental state examination was provided, the diagnosis – including differential diagnosis – and the treatment approach. One crucial point of her lecture was that treatment-resistant depressive disorders often occur in current medical practice, raising issues on differential diagnosis. Moreover, Prof. Ciobanu stated that these clinical cases must be addressed individually and highlighted the role of Cerebrolysin as adjuvant therapy for addressing treatment resistance.

**Brindusa Focsaneanu (Romania)**, Senior Lecturer at the Department of Psychiatry from the Faculty of Medicine at Titu Maiorescu University of Bucharest and the Head of the Psychiatry Department VIII within the Hospital Prof. Dr. Al. Obregia (Romania), presented the topics of depression and cognitive impairment, discussing therapeutic approaches for a case report on a 60-year-old woman experiencing a depressive episode with anxiety, behavioral and mood imbalance, somatic symptoms and others. She discussed the public health and epidemiological aspects of mental and neurological disorders in older adults, signalling the role of neurotrophic factors in the modulation of synaptic transmission and their part as a therapeutic strategy in certain neurological disorders. The fascinating presentation showcased a descriptive insight into the patient's pathway in the medical system. Dr. Focsaneanu compared the expected and actual outcomes and pinpointed the importance of cognitive testing, obtaining a detailed patient history, and implementing longitudinal follow-up. Moreover, Dr. Focsaneanu discussed the risks of dementia for older people with depression and the role of neurotrophic factors in aiding neurorecovery and the preservation of cognitive function.

**Andreea Szalontay (Romania)**, Associate Professor of Psychiatry at Grigore T. Popa University of Medicine and Pharmacy and Head of Clinical Psychiatry VI from the Socola Institute of Psychiatry in Iasi (Romania), presented a case of mild cognitive impairment after infection with SARS-CoV-2. Offering an epidemiological context on SARS-CoV-2 and cognitive decline, Prof. Szalontay mentioned the effects of COVID on bodily systems and highlighted the cognitive decline and the persistence of symptoms in patients with the infection. One point of her presentation was that the pandemic might contribute to the future increase in the world dementia burden. Her insightful and comprehensive case studies showcased the importance of multidimensional approaches and the effect of neurotrophic factors, such as Cerebrolysin, in the treatment of cognitive deterioration.

**Romeo Dobrin (Romania)**, Associate Professor of Psychiatry at Grigore T. Popa University of Medicine and Pharmacy and Head of Section IX at the Socola Institute of Psychiatry in Iasi (Romania), approached the efficacy of neurotrophic factors in neurocognitive disorders. Prof. Dobrin discussed the concept of senescence and dementia, presenting the stages of the disease and highlighting the symptoms and manifestations. Furthermore, based on a case report on mixed dementia, he highlighted the importance of comprehensive approaches, including mental state examination, Alzheimer's assessment, anamnesis, differential diagnosis and clinical data, showcasing the disease progression and presenting therapeutic objectives and further assessment. Finally, he outlined essential points on the management and perspectives of dementia, pinpointing the importance of a larger-picture approach, including research, medicine, and public health perspectives.

**Mihai Mutica (Romania)**, Psychiatrist at Elisabeta Doamna Psychiatry Hospital in Galati (Romania), ended the session with "Mild Cognitive Impairment in a Patient with Polymorphic Symptoms". Dr. Mutica showcased a case study with complex presentation and favorable outcomes, discussing the results of psychiatric examination, the effect of the treatment, and the management of the patient, pinpointing the importance of individualized approaches for anxiety affective symptoms presenting with cognitive impairment, advising the audience to take precautions when making assumptions about psychiatric disorders and their relation to future dementia.

The second session introduced **Virgil Enatescu (Romania)**, Habilitated Professor of Psychiatry at the Victor Babeș University of Medicine and Pharmacy in Timisoara (Romania), who presented "The Efficacy of Neurotrophic Factors in A Case Of Minor Cognitive Disorder Comorbid With Bipolar I Disorder And Sequelae Of Cerebral Infarctions". Prof. Enatescu discussed, based on a case study on bipolar disorder presenting with mild cognitive symptoms, the prognosis of bipolar disorders, genetic vulnerabilities, and comorbidities. Furthermore, he described the patient's pathway and the outcomes, pinpointing the impact of cognitive dysfunction on the psychosocial outcomes in patients with bipolar disorder. Cerebrolysin, as the potential aid in the treatment of a variety of debilitating neurological conditions, was also an important topic touched upon.

**Catalina Giurgi-Oncu (Romania)**, Senior Lecturer in Psychiatry at the Department of Neuroscience from Victor Babes University of Medicine and Pharmacy of Timisoara (Romania), presented "A significant improvement of affective symptoms and Theory of Mind abilities in a patient treated with neurotrophic factors for a Mild Cognitive Disorder". Dr. Giurgi-Oncu discussed mild cognitive impairment and the risk of developing further dementia, approached the impact of emotional health on social functioning, and glimpsed through the effect of depressive disorders on social interactions, highlighting the "Theory of Mind" concept encompassing affective and cognitive components. An exciting case study was presented, highlighting diagnostic criteria for mild cognitive impairment, epidemiology and risk factors, procedures of screening, evaluation, and early detection, and recommendations for follow-up and prognosis.

**Adriana Mihai (Romania)**, Associate Professor at the Psychiatric Department from the University of Medicine, Pharmacy, Science and Technology George Emil Palade in Targu Mures (Romania) and Director of the Centre of Psychotherapy and Personal Development (IPPD), showcased her presentation "Vascular neurocognitive disorder – presentation of a clinical case with language disturbances", highlighting cognitive disorders, especially vascular dementia, as a common cause of neurocognitive disorders post-Alzheimer's disease. Furthermore, Prof. Mihai presented epidemiological aspects and the characteristics of vascular dementia and introduced a fascinating clinical case presentation of acute psychotic disorder. Lastly, prof. Mihai discussed the importance of collecting comprehensive information on patients, especially on cases with less common manifestations, as the paradigm for treatment in psychiatry is a symptomatic one.

**Claudia Anghel (Romania)**, Psychiatrist at the Psychiatric Hospital Dr. Gh. Preda in Sibiu (Romania) and President of the NGO Psychiatric Hospital Association Dr. Gh. Preda, focused on "Electroconvulsive therapy – between necessity and stigma", discussing the mechanisms of electroconvulsive therapy and the impact of technological advancements while mentioning the possible complications and contraindications when using this therapy. For a better understanding, Dr. Anghel pinpointed the history of ElectroConvulsive Therapy (ECT) therapy in their hospital and the issues related to stigma and the portrayal of ECT in media, urging for improved education on this therapy. Lastly, in a case study on undifferentiated schizophrenia, the impact of ECT was highlighted. In the end, she invited the public to reflect on some aspects of ECT, including the avenues for changing societal perception of this medical technique.

In the form of discussions and final remarks, **Doina Cozman (Romania)** underlined the importance of showing great attention to mild cognitive impairment in people over 50 years of age, as this syndromologic diagnostic is chameleon-like, taking many possible forms and, in some cases, leading to dementia or depression, or even causing several psychiatric or somatic issues. Other critical points showcased were the relevance of the Montreal Cognitive Assessment (MoCA) test in cognitive assessment, the vital role of multidisciplinarity, and the interrelations of psychiatry and neurology. The parallel psychiatry session from the 17^th^ International Summer School of Neurology stands as an example of the importance of transdisciplinary perspectives in neurosciences.

